# Employees and the National Insurance Act

**Published:** 1912-05-18

**Authors:** 


### NATIONAL INSURANCE ACT DEPARTMENT.

### EMPLOYEES AND THE NATIONAL INSURANCE ACT.

### XI.—The Insurance of Nurses.

For the past ten weeks we have devoted our articles to a general consideration of the National Insurance Act. This general survey could easily have been extended, but as our immediate purpose is to assist our readers in taking full advantage of the benefits that are obtainable, we shall defer further general consideration of the Act for the present, and we commence with this issue to deal with its application to special classes of our readers.

A Nurse's Questions.

At the close of our article last week we announced the receipt of a letter from a nurse, in which information was asked concerning her position under the Act. The letter raises so many points of general interest that we make no apology for selecting her case as an illustration of many others. Our correspondent says: "I am a hospital nurse, on the outdoor staff of a nursing home, receiving salary on the co-operative system. I am wondering how I do when the payments are due. Do I apply for the cards or forms, and to whom? Do I come under casual labour—the nursing home does not pay me a regular salary; they get so much per cent. from each case they send me to? Do the people whom I am nursing pay my weekly contributions of 3d. and myself 3d.? Say I should go to a case, perhaps on a Tuesday, and not stay the whole week, who then pays my employer's 3d.? Or should I go perhaps on the Sunday and stay until Monday night or Tuesday, is it due to the employer to whom I have been to pay for that week? And is my contribution and also the contribution of an employer due to be paid while I am not at a case—not meaning my holidays, but the waiting time between cases? If not busy sometimes this is a week, or may be more; at such times I am not receiving any salary, and have full hostel expenses. Would it be easier and a benefit for me to join a society? I see that the Royal National Pension Fund for Nurses is starting a National Insurance Society. I am not a member of the Pension Fund, but I suppose I could join the Insurance Society. If I did join it do I pay my contributions to the Insurance Society or still pay weekly payments, and would the Society pay my benefits in case of sickness or accident? I am already in an insurance society, paying a yearly premium for benefit in case of accident or certain sickness—infectious diseases. Will this make any difference to my receiving benefit of the National Insurance in case of sickness or accident? I should be glad of information about the Nurses' National Insurance Society. I am really anxious to understand the Act."

Our Reply.

These numerous inquiries are all perfectly natural, and explicit replies can be given to most of them, but, like the Chancellor of the Exchequer in the House the other day, when pressed with difficult questions on the Act, we find it convenient to ask further questions, in the first instance, rather than answer directly those propounded. The first question we have to ask is, Does our correspondent come within the scope of the compulsory provisions of the Act?

This is the main question, and the answer needs consideration. The nursing profession is not mentioned specifically in the Act, either as included or excluded, and we must therefore proceed to examine the sections which will enable us to decide. Inclusion depends first upon age, which must not be less than sixteen nor more than sixty-five on entry into insurance. In this respect there is little doubt as to the application of the Act to our correspondent. In the next place she must be employed "within the meaning of the Act" in order to be entitled to benefits, and the conditions governing this are set out in the First Schedule. The first subsection defining such employment is the one appropriate to the case, and the answer depends upon its interpretation. The subsection reads: "Employment in the United Kingdom under any contract of service or apprenticeship, written or oral, whether expressed or implied, and whether the employed person is paid by the employer or some other person, and whether under one or more employers, and whether paid by time or by the piece or partly by time and partly by the piece, or otherwise, or, except in the case of a contract of apprenticeship, without any money payment."

Who is the Employer?

There is no difficulty in deciding that the nurse is an employed person in the United Kingdom, but there are serious difficulties in deciding who is the employer, and in stating definitely who are the parties to the contract of employment. In general a contract of service exists if the servant is bound (*a*) personally to execute work (*b*) for another (*c*) exclusively, (*d*) in return for wages or other remuneration (*e*) acting under the orders of that other. The nurse's services are essentially personal, and they are undoubtedly for another. By the special provision of the Act employment need not be exclusive, so that the general definition does not apply in this respect. Remuneration is received by the nurse for the services she renders, and it is most certainly her duty to act according to her orders. It should be noted, however, that the payment of wages or other remuneration does not of itself create the relation of master and servant. The contract of service may be between A., as employer, and B., as employee, while C. pays for the services rendered.

In a matter of so much complexity we cannot lay down any hard-and-fast rule, but it appears that as the nurse presumably acts under the orders of the nursing home, and not under the orders of her patients, although she receives no money payment from the home, she is employed by the home and not by the patient. We are confirmed in this view by the consideration that in many cases the nurse is not personally engaged by the patients, but a request is sent to the home for a nurse to be supplied, and she obeys the orders of the matron in attending the case. The matter will, of course, have to be definitely decided, and the authority whose judgment is final are the Insurance Commissioners. There is little doubt that the question will be brought to the notice of the Commissioners by the Nurses' National Insurance Society, if it has not done so already.

### Is the Employment Casual?

If our answer to the first question be correct, the inquiry as to whether the employment is casual would seem to be readily answered in the negative. If, however, it should be decided that the patients are the employers there would seem to be greater room for doubt. The dictionary meaning assigned to the word casual is "arising from chance, irregular," and it would seem that there are good grounds for assuming that the employment of a nurse for a few days only would be considered irregular or as arising from chance. If the employment is decided to be casual as it is not for the purpose of the employer's business, it would exempt the employer from the payment of compulsory contributions.

There are other conditions to be fulfilled if the nurse is to be compulsorily insured. Her rate of remuneration, for instance, must not be more than £160 per annum, and it is possible that she may hold a certificate of exemption on the ground that she is in receipt of a pension or income of the annual value of not less than £26—not dependent upon her personal exertions—or that she is mainly dependent upon some other person.

### How the Contributions are Paid.

Assuming, then, that the home is responsible as the employer, and that the nurse is not exempted from insurance, it will be the duty of the matron or other person in charge to pay the weekly contributions of 6d. on the nurse's behalf. Of this sum 3d. is the employer's contribution, and may not be recovered from the nurse, while the remaining 3d., being the employee's contribution, may be so recovered. As no wages are paid in the case in question, recovery may be made otherwise than by deduction from the wages. That is to say, the home will have the right to claim from the nurse not only the stipulated percentage of her fees, but a further sum of 3d. per week to cover her National Insurance contributions. Only one contribution will be required for each week or part of a week in which she is employed, so that attendance on two patients in one week will not necessitate two contributions being paid. On the other hand, the employer is not responsible for the payment of the contributions in any week during which no services are rendered and no remuneration is paid. This would seem to exempt the home from responsibility of paying the contributions for any calendar week (that is the period from midnight on one Sunday to midnight on the following Sunday) in which the nurse was not required to attend a patient. Although the home does not pay the contribution in such circumstances, the payment will, nevertheless, become due, unless the nurse was herself incapacitated and in receipt of sickness or disablement benefit, and if the nurse does not pay the full contribution of 6d. herself she will be in arrear to that extent, and suffer reductions of benefit on the lines indicated last week.

### How to Join a Society.

It will be immensely to the advantage of the nurse to join a society instead of contributing through the Post Office, and we have no hesitation in recommending our correspondent to communicate as soon as possible with the Secretary of the Nurses' National Insurance Society, 15 and 16 Buckingham Street, Strand, London, W.C. A form of application for membership will then be sent, and in due course, if this is satisfactorily completed, the nurse will be admitted and will be supplied with a book or card to which the stamps representing the contributions will be attached. The name of the society will not appear on the card, as it is not essential for the employee to disclose to the employer the society which she has elected to join. When the card has been filled it will have to be returned to the society, and a fresh one will be issued to take its place. The society will then proceed to make the necessary claim on the Insurance Commissioners for the funds represented by the stamps to be duly credited to its account.

As soon as the prescribed waiting periods have elapsed the nurse will be entitled to the benefits provided by the Act, which will be administered through the society. We need not here specify the benefits, nor the conditions under which they may be claimed, but we would point out that they are much more comprehensive than those that appear to be obtainable under the policy that our correspondent already holds. Insurance under the National Scheme does not preclude the insured person from supplementing the benefits by others which are paid for separately, but it is quite likely that the insurance company may require a declaration, before renewing the policy, that the total benefit receivable from all sources during sickness does not exceed the sum usually received in wages.

No society has yet received the formal approval of the Commissioners, and pending such approval we are unable to furnish full details of any society. The Nurses' National Insurance Society has, we understand, applied for approval, and in due course its rules, as approved, will be available on application being made to the secretary.

---

Note.—Questions and Correspondence on all doubtful points are invited, and should be addressed to the Insurance Editor, The Hospital, 28 Southampton Street, Strand.

